# Quality of life in adults from a rural area in Southern Brazil: a population-based study

**DOI:** 10.11606/S1518-8787.2018052000261

**Published:** 2018-09-13

**Authors:** Caroline Cardozo Bortolotto, Christian Loret de Mola, Luciana Tovo-Rodrigues

**Affiliations:** I Universidade Federal de Pelotas. Faculdade de Medicina. Programa de Pós-Graduação em Epidemiologia. Pelotas, RS, Brasil; II Universidade Federal de Pelotas. Faculdade de Enfermagem. Pelotas, RS, Brasil

**Keywords:** Adult, Quality of Life, Socioeconomic Factors, Gender and Health, Rural Population, Adulto, Qualidade de Vida, Fatores Socioeconômicos, Gênero e Saúde, População Rural

## Abstract

**OBJECTIVE:**

To analyze the quality of life and its determinants in a population living in a rural area.

**METHODS:**

This is a population-based, cross-sectional study with individuals aged 18 years or over from the rural area of Pelotas, State of Rio Grande do Sul, Brazil. We evaluated quality of life using the WHOQOL-BREF, which has four domains (physical, psychological, social relations, and environment) and two questions: overall quality of life and satisfaction with health. We considered as independent variables the demographic, socioeconomic, and health variables. We evaluated the associations using linear regression in the four domains and ordinal logistic regression in the two general questions on quality of life and satisfaction with health.

**RESULTS:**

The sample consisted of 1,479 individuals. The prevalence of the perception of overall very poor quality of life and dissatisfaction with health were 22.5% and 26.3%, respectively. Individuals who were older (p < 0.001), non-white (p = 0.004), with lower education level (p < 0.001), poorer (p = 0.001), and who had always lived in the rural area (p = 0.049) were less likely to have a better perception of overall quality of life. As for satisfaction with health, women (p = 0.001), older individuals (p = 0.001), those unemployed (p = 0.023), and those with diseases were less likely to report higher satisfaction with health. For the four domains evaluated, the results were consistent with those observed for the general questions.

**CONCLUSIONS:**

The most relevant aspects that negatively defined the quality of life of the population were being a woman, older, non-white, having a low income, having a lower education level, having always lived in the rural area, being unemployed, and having a disease. Given that they are significant factors as determinants of health, these results suggest that quality of life is an issue that should be placed among health needs, especially regarding the most vulnerable groups in rural areas.

## RESUMO

**OBJETIVO:**

Analisar a qualidade de vida e seus determinantes em uma população residente na zona rural.

**MÉTODOS:**

Estudo transversal de base populacional com indivíduos de 18 anos ou mais, da zona rural de Pelotas, Sul do Brasil. A qualidade de vida foi avaliada pelo WHOQOL-BREF, composto por quatro domínios (físico, psicológico, relações sociais e meio ambiente), e duas perguntas: qualidade de vida global e satisfação com a saúde. Variáveis demográficas, socioeconômicas e de saúde foram consideradas entre as variáveis independentes. As associações foram avaliadas por regressão linear nos quatro domínios e por regressão logística ordinal nas duas perguntas gerais de qualidade de vida e satisfação com a saúde.

**RESULTADOS:**

A amostra foi composta por 1.479 indivíduos. As prevalências de percepção de qualidade de vida global muito ruim e insatisfação com a saúde foram, respectivamente, 22,5% e 26,3%. Indivíduos mais velhos (p < 0,001), com cor da pele não branca (p = 0,004), com menor escolaridade (p < 0,001), mais pobres (p = 0,001) e que residiram a vida toda na zona rural (p = 0,049) apresentaram menor chance de ter melhor percepção de qualidade de vida global. Quanto à satisfação com a saúde, as mulheres (p = 0,001), os mais velhos (p = 0,001), os desempregados (p = 0,023) e aqueles portadores de doenças tiveram menor chance de relatarem maior satisfação com a saúde. Para os quatro domínios avaliados, os resultados foram consistentes com os observados para as perguntas gerais.

**CONCLUSÕES:**

O fato de ser mulher, mais velho, não ser branco, ter baixa renda, ter menor escolaridade, residir a vida toda na zona rural, estar desempregado e portar alguma doença foram os aspectos mais relevantes para definir negativamente a qualidade de vida da população. Tendo em vista que são fatores significativamente importantes como determinantes da saúde, estes resultados sugerem que a qualidade de vida é um tema que deve ser colocado entre as necessidades de saúde, principalmente com relação aos grupos mais vulneráveis das áreas rurais.

## INTRODUCTION

Quality of life (QOL) covers the aspects of physical and mental health, social relationships, and personal beliefs, as well as environmental characteristics^1^. In addition, it has been growing in importance in the evaluation of therapeutic interventions and services and in the daily health care practices in the health area^2^.

Brazilian rural populations are different from urban populations in a series of factors that influence quality of life, such as demographic and socioeconomic aspects, with lower education level^3^ and monthly average income^4^. Regarding health indicators, the results in the literature are contradictory^5–8^. However, the lower access to health services is reported consistently, and the low coverage by health plans has already been described in these regions^6^ ^,^ ^8^. Furthermore, differences related to the physical and social environments, such as air quality, noise pollution, and lifestyle have been highlighted in the literature^9^. Specifically in Brazil, the National Health Survey (PNS) has indicated that the prevalence of smoking and physical activity at work are higher in rural than in urban inhabitants^6^. It has also verified that exposure to pesticides reaches a significant portion of the population^7^. In addition, there is considerable data on the mental health of rural residents where the frequency of self-reported depression is approximately 6% and more prevalent in women^6^.

In 2010, approximately 15% of the Brazilian population lived in rural areas, which is similar to the amount described for the state of Rio Grande do Sul at the time^10^. However, knowledge about the health and QOL aspects of this considerable portion of the population is scarce. The objective of this study was to characterize the QOL and factors that may be associated with the health of an adult population living in a rural area of Southern Brazil.

## METHODS

This population-based, cross-sectional study is part of a larger study named “Evaluation of the Health of Adults Living in the Rural Area of the City of Pelotas, State of Rio Grande do Sul, Brazil” (“*Avaliação da Saúde de Adultos Residentes na Zona Rural do Município de Pelotas, RS*”), carried out between January and July 2016. We evaluated a representative sample of the adult population (aged 18 years or over) living in the rural area of the city of Pelotas, State of Rio Grande do Sul, Brazil. This region has eight districts and 50 census tracts and comprises 7% of the population of the city^11^.

We used a multistage cluster sampling. A total of 24 census tracts were drawn systematically, proportional to the number of permanent households in each district. In total, 720 households were visited (30 per tract). Individuals institutionalized at the time of the research or if they had some cognitive impairment that made them unable to answer the questionnaire were excluded from the sample. More details on the methodology can be obtained in the methodological article^12^.

The QOL was evaluated with the WHOQOL-BREF instrument^1^. This instrument has 26 questions and two of them separately evaluate overall QOL and satisfaction with health. The remaining 24 questions encompass four domains: physical (pain and discomfort, energy and fatigue, sleep and rest, mobility, activities of daily living, dependence on medication or treatments, and ability to work), psychological (positive feelings, thinking, learning, memory, and concentration, self-esteem, body image and appearances, negative feelings, spirituality, religiosity, and personal beliefs), social relations (personal relationships, social support, and sexual activity), and environment [physical security and protection, home environment, financial resources, health and social care, availability and quality, opportunity to acquire new information and skills, participation in recreation or leisure opportunities, physical environment (pollution, noise, traffic, climate), transport]. These questions represent each of the 24 aspects that make up the original instrument (WHOQOL-100)^13^. We used the period of two weeks before the interview for all of them. The answers to all questions range from one to five in a Likert scale. We derived and standardized the individual scores for each one of the domains on a scale from one to 100, according to the protocol suggested by the WHOQOL Group^13^. For the analyses, we grouped the original five categories of responses into three: very good; good; fair, poor, or very poor for the perception of overall QOL, and very satisfied; satisfied; fair, dissatisfied, or very dissatisfied for the perception of health.

We evaluated the association between determinant variables and outcomes based on a hierarchical conceptual model with four levels. At the first level, we included the following variables: sex (male, female), age (18 to 24, 25 to 39, 40 to 59, 60 years or over), and self-reported race (white, non-white); in the second level, we used: education level (zero to four, five to eight, nine to eleven, twelve or more years of study), index of goods (quintiles), employment (no, yes), and number of residents (of any age group) of the domicile (one, two, three, four, five or more); in the third level, we used: living with a partner (no, yes) and percentage of time living in the rural area (less than 50%, between 50 and 99%, 100%). At the fourth level, we included the variables of self-reported diagnosis of:)high blood pressure (HBP), diabetes, high cholesterol, cardiovascular diseases (heart failure or angina), and respiratory (asthma or bronchitis) and rheumatic diseases (arthritis, rheumatism, or osteoarthritis) (yes, no).

We obtained the index of goods from the principal component analysis containing 22 questions asked to the head of the family, who evaluated the amount of goods in the household at the time: piped water, vacuum cleaner, washing machine, dryer, dishwasher, DVD, VCR, refrigerator, microwave, computer (notebook or netbook), television, radio, air conditioner, cable TV or internet, car or motorcycle. Furthermore, it also contained the number of toilets/bathrooms, number of rooms used to sleep, and whether they employed a domestic worker. This variable of index of goods was analyzed in quintiles, ranging from the poorest quintile (1st) to the richest quintile (5th).

We used the survey (*svy*) command in all analyses, considering the effect of cluster sampling. In addition, we used a weighting considering the percentage of households sampled in relation to the number of permanent households of each district according to IBGE data^11^.

For the four domains of QOL, we performed the crude and adjusted analyses by linear regression. We presented the average score (crude), the adjusted coefficients (β) and the 95% confidence interval (95%CI). For both questions of overall QOL and satisfaction with health, we performed the crude and adjusted analyses by ordinal logistic regression, obtaining estimates of odds ratio (OR) and 95%CI. For these analyses, we obtained estimates of the probability of going from one category of perception of QOL and satisfaction with health to the subsequent category, considering the directionality from the best to the worst category of response in all cases. We used the Brant test to evaluate the assumption of proportionality of the OR among the categories of outcome. We performed adjusted analyses according to the conceptual hierarchical level model, in which the variables were adjusted for all variables of the same level, as well as for those of the previous level in the model. This method was repeated for the other levels. We considered the level of statistical significance of 0.05 for associations between variables and outcomes. We calculated the Kappa value based on the question “Do you know how to read or write?”, indicating 76% of repeatability.

The project was approved by the Research Ethics Committee of the Faculdade de Medicina of the Universidade Federal de Pelotas (Process 1.363.979). All research participants signed an informed consent.

## RESULTS

Of the 1,697 individuals eligible for the study, 1,519 were interviewed and 1,479 were included in this study. The value of losses and refusals was 10.5%. Most of the interviewees were female (51.6%), white (85.3%), had up to eight years of complete studies (75%), and reported having a job at the time (61%). Approximately 40% of them were aged between 40 and 59 years and 26.2% lived in households that had three persons. The most prevalent diseases were high cholesterol and rheumatic diseases, 14.9% and 19.3%, respectively. Regarding the perception of overall QOL and health, 22.5% reported fair, poor, or very poor QOL and 26.3% were dissatisfied with their health (Table 1).

**Table 1:** Characterization of the sample according to socioeconomic, demographic, and behavioral characteristics. Pelotas, State of Rio Grande do Sul, Brazil, 2016. (n = 1,479)

Variable	n	%
Sex		
Male	716	48.4
Female	763	51.6
Age (full years)		
18–24	170	11.5
25–39	341	23.1
40–59	587	39.6
≥ 60	381	25.8
Race		
White	1,262	85.3
Black	88	6.0
Brown	100	6.8
Yellow	21	1.4
Indigenous	8	0.5
Education level (full years)		
0–4	552	37.5
5–8	553	37.5
9–11	301	20.5
≥ 12	66	4.5
Index of goods^a^ (quintiles)		
1st (poorest)	295	20.0
2nd	293	20.0
3rd	295	20.1
4th	295	20.1
5th (richest)	291	19.8
Number of residents		
1	92	6.3
2	372	25.3
3	386	26.2
4	300	20.4
≥ 5	322	21.9
Employment		
No	577	39.0
Yes	902	61.0
Living with a partner		
No	421	28.5
Yes	1,058	71.5
% of time living in the rural area		
Less than 50%	276	18.7
50% to 99%	207	14.0
100%	996	67.3
High blood pressure		
No	976	66.1
Yes	501	33.9
Diabetes		
No	1,333	90.2
Yes	144	9.8
High cholesterol		
No	1,191	80.7
Yes	284	19.3
Cardiovascular diseases^b^		
No	1,380	93.8
Yes	92	6.3
Respiratory diseases		
No	1.331	90.1
Yes	146	9.9
Rheumatic diseases		
No	1,256	85.1
Yes	220	14.9
Perception of overall quality of life		
Very poor/Poor/Fair	333	22.5
Good	456	64.6
Very good	190	12.9
Satisfaction with health		
Very dissatisfied/Dissatisfied/Fair	289	26.3
Satisfied	942	63.7
Very satisfied	147	10.0

^a^ Index of goods by principal components.

^b^ Variable with most missing data.

Table 2 presents the estimates of association between the independent variables and the four domains of QOL evaluated based on the hierarchical theoretical model. For the physical domain, women presented, on average, approximately five points less for QOL than men. As the age group increased, we observed a linear trend of worse QOL in this domain (p < 0.001), so that individuals aged 60 years or over presented, on average, 11.0 points less than younger individuals. Individuals with lower education level (zero to four years, five to eight years) reported worse QOL than those with nine to eleven years and twelve years or more. As for the index of goods, the poorest quintile also presented a significant difference in relation to the richest quintile. Finally, employed individuals had, on average, 4.0 points more of QOL than those not employed. All diseases assessed were associated with lower QOL scores in the physical domain. Rheumatic diseases had the worst average score (95%CI -10.9– -6.82).

**Table 2:** Average quality of life scores and their coefficients (β) adjusted according to the socioeconomic, demographic, and behavioral variables. Pelotas, State of Rio Grande do Sul, Brazil, 2016.

Variable	Domain 1 (physical)		Domain 2 (psychological)		Domain 3 (social relations)		Domain 4 (environment)	
			
	Average (SD)	β^a^ (95%CI)	Average (SD)	ββ^a^ (95%CI)	Average (SD)	ββ^a^ (95%CI)	Average (SD)	ββ^a^ (95%CI)
Sex		p < 0.001^c^		p < 0.001^c^		p = 0.171^c^		p = 0.005^c^
Male	77.9 (13.3)	Ref.	74.9 (11.9)	Ref.	76.4 (13.2)	Ref.	65.4 (12.3)	Ref.
Female	73.0 (15.3)	-4.90 (-5.99– -3.80)	70.1 (14.3)	-4.87 (-6.05– -3.70)	75.4 (13.5)	-1.06 (-2.60–0.49)	63.6 (13.2)	-1.80 (-3.02– -0.59)
Age (full years)		p<0.001^d^		p = 0.001^d^		p < 0.001^d^		p = 0.051^c^
18–24	82.1 (11.2)	Ref.	75.8 (13.0)	Ref.	79.3 (14.5)	Ref.	67.1 (12.9)	Ref.
25–39	78.1 (14.2)	-3.92 (-7.06– -0.78)	72.9 (13.4)	-2.70 (-5.13– -0.28)	77.2 (14.5)	-2.00 (-5.70–1.69)	63.9 (14.1)	-3.13 (-6.64–0.38)
40–59	74.8 (14.32)	-7.05 (-9.00– -5.10)	72.3 (13.7)	-3.19 (-5.60– -0.77)	75.6 (12.6)	-3.43 (-6.10– -0.77)	63.7 (12.1)	-3.41 (-5.87– -0.94)
≥ 60	70.8 (15.0)	-11.0 (-13.3– -8.75)	70.6 (12.8)	-4.90 (-7.19– -2.60)	73.8 (12.5)	-5.20 (-7.87– -2.54)	64.9 (12.5)	-2.21 (-5.14–0.73)
Race		p = 0.302^c^		p = 0.831^c^		p = 0.759^c^		p = 0.157^c^
White	75.4 (14.2)	Ref.	72.4 (13.2)	Ref.	75.8 (12.9)	Ref.	64.9 (12.6)	Ref.
Other	75.0 (16.4)	-1.10 (-3.26– - 1.06)	72.3 (14.5)	-0.28 (-2.95–2.39)	76.5 (15.9)	0.30 (-1.687–2.27)	61.7 (13.9)	-1.95 (-4.69–0.80)
Education level (full years)		p = 0.020^c^		p < 0.001^d^		p = 0.331^c^		p = 0.225^c^
0–4	71.9 (15.6)	Ref.	69.6 (13.8)	Ref.	74.4 (12.9)	Ref.	63.0 (12.7)	Ref.
5–8	75.5 (13.6)	1.17 (-0.78–3.13)	72.6 (12.9)	2.03 (0.46–3.60)	75.4 (13.4)	-0.46 (-1.91–1.00)	63.9 (12.3)	0.18 (-1.09–1.45)
9–11	80.6 (12.4)	4.19 (1.71–6.66)	75.9 (12.2)	4.08 (1.34–6.81)	78.4 (13.3)	0.63 (-1.85–3.11)	67.0 (13.2)	1.90 (-0.28–4.07)
≥ 12	80.1 (13.9)	4.18 (-0.28–8.63)	78.8 (12.1)	6.81 (2.95–10.68)	81.1 (14.4)	3.26 (-2.03–8.54)	69.4 (13.8)	2.63 (-1.29–6.55)
Index of goods^b^ (quintiles)		p = 0.089^c^		p = 0.001^d^		p = 0.018^c^		p = 0.002^c^
1st (poorest)	72.2 (15.7)	Ref.	69.7 (14.6)	Ref.	73.5 (13.7)	Ref.	60.4 (14.0)	Ref.
2nd	75.4 (14.2)	2.23 (-0.36–4.82)	70.9 (13.2)	0.68 (-1.03–2.39)	75.3 (13.4)	1.77 (-0.25–3.79)	63.2 (12.6)	2.88 (0.33–5.43)
3rd	74.4 (15.5)	1.02 (-1.35–3.40)	71.9 (13.5)	1.55 (-0.86–3.96)	76.4 (13.2)	3.39 (0.54–6.23)	63.7 (12.3)	3.40 (0.91–5.89)
4th	75.7 (13.5)	1.84 (-1.41–5.09)	73.6 (13.1)	2.88 (0.32–5.43)	76.3 (12.2)	3.02 (0.61–5.42)	65.9 (11.8)	5.14 (2.20–8.09)
5th (richest)	79.3 (12.6)	4.68 (1.49–7.87)	69.6 (7.87)	4.34 (1.73–6.95)	69.2 (28.1)	4.97 (2.49–7.45)	69.0 (11.7)	7.66 (4.70–10.63)
Number of residents		p = 0.272^c^		p = 0.182^c^		p = 0.001^d^		p = 0.468^c^
1	74.8 (13.7)	Ref.	70.3 (14.5)	Ref.	75.2 (13.9)	Ref.	65.1 (13.8)	Ref.
2	74.0 (13.7)	-2.70 (-6.41–0.99)	73.0 (13.5)	0.86 (-3.28–5.00)	75.9 (12.7)	-0.86 (-4.54–2.82)	63.9 (13.1)	-2.77 (-7.24–1.69)
3	75.8 (10.7)	-3.82 (-7.63– -0.01)	72.6 (14.3)	-1.26 (-5.14–2.62)	76.9 (13.6)	-1.47 (-5.14–2.19)	64.2 (12.9)	-3.60 (-8.47–1.27)
4	76.6 (13.0)	-3.37 (-6.96–0.22)	72.7 (12.2)	-1.09 (-4.85–2.67)	76.6 (11.3)	-2.24 (-6.01–1.53)	64.2 (12.5)	-3.96 (-8.82–0.89)
≥ 5	75.7 (14.6)	-4.54 (-8.93– -0.15)	71.9 (13.0)	-1.91 (-5.76–1.93)	74.7 (14.4)	-4.35 (-8.56– -0.13)	65.5 (12.3)	-2.97 (-7.58–1.63)
Employment		p < 0.001^c^		p = 0.034^c^		p = 0.050^c^		p = 0.064^c^
No	71.5 (16.0)	Ref.	70.1 (14.5)	Ref.	74.2 (13.5)	Ref.	63.0 (13.4)	Ref.
Yes	77.9 (13.0)	4.03 (2.03–6.04)	73.9 (12.4)	1.76 (0.14–3.37)	77.0 (13.1)	1.72 (-0.01–3.43)	65.3 (12.4)	1.80 (-0.11–3.71)
Living with a partner		p = 0.825^c^		p = 0.019^c^		p = 0.249^c^		p = 0.621^c^
No	75.9 (14.8)	Ref.	71.5 (14.0)	Ref.	75.4 (13.5)	Ref.	64.8 (13.2)	Ref.
Yes	75.2 (14.4)	0.16 (-1.33–1.65)	72.8 (13.1)	1.79 (0.32–3.28)	76.1 (13.3)	1.13 (-0.85–3.11)	64.3 (12.7)	-0.43 (-2.21–1.35)
% of time living in the rural area		p = 0.698^c^		p = 0.935^c^		p = 0.433^c^		p = 0.767^c^
Less than 50%	76.1 (15.9)	Ref.	73.5 (14.8)	Ref.	77.1 (14.7)	Ref.	64.2 (13.9)	Ref.
50% to 99%	73.7 (14.7)	-1.02 (-3.83–1.80)	71.4 (14.0)	-0.42 (-2.88–2.04)	74.7 (13.4)	-1.76 (-4.49–0.98)	63.9 (13.2)	0.63 (-1.43–2.70)
Up to 100%	75.5 (14.1)	-0.14 (-2.71–2.42)	72.3 (12.9)	-0.02 (-2.29–2.24)	75.8 (12.9)	-0.50 (-3.04–2.04)	64.6 (12.4)	0.71 (-1.39–2.80)
High blood pressure		p = 0.005^c^		p = 0.074^c^		p = 0.449^c^		p = 0.746^c^
No	77.8 (14.3)	Ref.	73.8 (13.3)	Ref.	76.7 (13.8)	Ref.	64.7 (12.9)	Ref.
Yes	70.7 (10.7)	-2.50 (-4.18– -0.83)	69.8 (13.3)	-1.53 (-3.22–0.16)	74.4 (12.3)	-0.51 (-1.89–0.87)	63.8 (12.7)	0.26 (-1.40–1.93)
Diabetes		p = 0.028^c^		p = 0.021^c^		p = 0.396^c^		p = 0.054^c^
No	76.1 (14.3)	Ref.	73.0 (13.1)	Ref.	76.2 (13.3)	Ref.	64.7 (12.7)	Ref.
Yes	16.6 (10.7)	-3.20 (-4.18– -0.83)	67.0 (16.7)	-3.36 (-6.16– -0.56)	73.4 (14.0)	-0.94 (-3.20–1.31)	61.7 (13.5)	-2.21 (-4.47– - 0.05)
High cholesterol		p = 0.005^c^		p = 0.014^c^		p = 0.040^c^		p = 0.035^c^
No	76.9 (14.3)	Ref.	73.3 (12.9)	Ref.	76.5 (13.2)	Ref.	64.9 (12.7)	Ref.
Yes	69.1 (10.7)	-2.61 (-4.36– -0.87)	68.7 (14.9)	-2.26 (-4.01– -0.51)	73.5 (13.3)	-1.88 (-3.68– -0.09)	62.6 (13.1)	-1.76 (-3.39– -0.13)
Cardiovascular diseases^e^		p = 0.022^c^		p = 0.785^c^		p = 0.428^c^		p = 0.689^c^
No	76.2 (10.7)	Ref.	72.7 (13.2)	Ref.	76.1 (13.5)	Ref.	64.6 (12.8)	Ref.
Yes	63.8 (17.9)	-4.15 (-7.64– -0.66)	67.7 (16.7)	0.34 (-2.21–2.89)	73.5 (11.7)	0.98 (-1.54–3.51)	62.3 (12.7)	0.60 (-2.44–3.64)
Respiratory diseases		p = 0.003^c^		p = 0.079^c^		p = 0.574^c^		p = 0.029^c^
No	75.8 (14.4)	Ref.	72.7 (13.3)	Ref.	76.0 (13.3)	Ref.	64.7 (12.8)	Ref.
Yes	71.1 (15.5)	-3.54 (-5.75– -1.33)	69.7 (14.2)	-2.25 (-4.78–0.28)	75.5 (13.9)	-0.46 (-2.15–1.22)	62.0 (12.9)	-2.48 (-4.67– -0.28)
Rheumatic diseases		p < 0.001^c^		p < 0.001^c^		p = 0.014^c^		p = 0.002^c^
No	77.2 (14.3)	Ref.	73.4 (13.0)	Ref.	76.5 (13.5)	Ref.	64.9 (12.8)	Ref.
Yes	65.2 (10.7)	-8.84 (-10.9– -6.82)	66.9 (14.2)	-5.08 (-7.12– -3.04)	72.8 (12.4)	-2.63 (-4.68– -0.59)	62.0 (12.9)	-2.69 (-4.25– -1.13)

^a^ Multiple linear regression adjusted for sex, age, race, education level.

^b^ Index of goods by principal components, employment, number of residents in the household, living with a partner, percentage of time living in the rural area, work, and number of residents.

^c^ P-value for heterogeneity.

^d^ P-value for linear trend.

^a^ Variable with most missing data.

Regarding the psychological domain, women reported, on average, 5.0 points less than men. Regarding age, we observed worse QOL with increasing age (p = 0.001). Older individuals had 4.9 points less for QOL than younger persons. As for education level, there was an improvement in QOL with increasing study years (p < 0.001). Individuals with a lower education level had, on average, 7.9 points less for QOL than those with a higher education level. As for index of goods, the poorest quintile presented, on average, 3.6 points less for QOL than the richest quintile. Being employed (p = 0.034) and living with a partner (p = 0.019) were associated with the highest average QOL. Individuals who reported having diabetes, high cholesterol, or rheumatic diseases had worse QOL averages than the reference groups (Table 2).

For the domain of social relations, we observed a linear trend of worse QOL averages with increasing age (p < 0.001). Older adults presented, on average, 5.2 points less than younger individuals. In relation to the index of goods, the poorest quintile had a lower QOL score than the richest quintile (Table 2). As the number of residents per household increased, the average QOL scores were worse (p = 0.001). The individuals who were employed at the time of the interview reported, on average, 1.7 points more than their reference category (p = 0.050). In relation to diseases, having high cholesterol or rheumatic diseases were associated with worse QOL scores (Table 2).

For the environmental domain, women had, on average, 1.8 points less than men. In relation to age, only the category between 40 and 59 years showed a statistically significant decrease in QOL points compared to younger individuals. The richest quintile had a better QOL than the poorest quintile (7.7 points on average). The presence of high cholesterol and respiratory and rheumatic diseases was associated with worse QOL scores. Rheumatic diseases again had the worst score (95%CI -4.67– -0.28).

The variables of race and the percentage of time living in the rural area were not associated with any of the four domains.

Considering the two general questions (Table 3), we observed that, as age increased, the chances of having worse QOL (going from very good to good and from good to fair, poor, or very poor) also increased (p = 0.001). Non-white individuals were 1.7 times more likely to report categories of worse QOL (95%CI 1.20–2.28). In addition, we observed greater protection for the reporting of worse QOL in the richest quintiles and in the higher education levels. For the individuals who lived their entire lives in the rural area, the chance of reporting a worse perception of overall QOL was 35% higher than for those who lived less than half of their lives (p = 0.049). In the presence of diseases, the chance of having a worse QOL was higher among individuals with high cholesterol (95%CI 1.04–1.85).

**Table 3:** Odds ratio (OR) adjusted according to the socioeconomic, demographic, and behavioral variables. Pelotas, State of Rio Grande do Sul, Brazil, 2016.

Variable	Perception of overall quality of life^f^		Satisfaction with health^f^	

	(I) Very good (II) Good (III) Fair, poor, or very poor		(I) Very satisfied (II) Satisfied (III) Fair, dissatisfied, or very dissatisfied	

	Crude OR (95%CI)	Adjusted OR^a^ (95%CI)	Crude OR (95%CI)	Adjusted OR^a^ (95%CI)
Sex	p = 0.744^c^	p = 0.925^c^	p = 0.001^c^	p = 0.001^c^
Male	Ref.	Ref.	Ref.	Ref.
Female	1.04 (0.83–1.29)	1.01 (0.80–1.27)	1.51 (1.21–1.88)	1.50 (1.19–1.89)
Age (full years)	p < 0.001^d^	p < 0.001^d^	p = 0.001^d^	p = 0.001^d^
18–24	Ref.	Ref.	Ref.	Ref.
25–39	1.92 (1.21–3.06)	1.90 (1.19–3.03)	1.71 (1.19–2.47)	1.70 (1.18–2.44)
40–59	2.44 (1.80–3.32)	2.52 (1.84–3.46)	2.04 (1.30–3.22)	2.06 (1.32–3.23)
≥ 60	3.33 (2.34–4.75)	3.47 (2.47–4.90)	2.24 (1.55–3.24)	2.26 (1.56–3.27)
Race	p < 0.001^c^	p = 0.004^c^	p = 0.490^c^	p = 0.308^c^
White	Ref.	Ref.	Ref.	Ref.
Other	1.51 (1.09–2.08)	1.65 (1.20–2.28)	1.12 (0.81–1.55)	1.18 (0.85–1.63)
Education level (full years)	p < 0.001^d^	p < 0.001^d^	p = 0.002^d^	p = 0.124^d^
0–4	Ref.	Ref.	Ref.	Ref.
5–8	0.60 (0.47–0.76)	0.71 (0.54–0.94)	0.85 (0.66–1.09)	0.99 (0.76–1.28)
9–11	0.29 (0.21–0.40)	0.48 (0.33–0.70)	0.61 (0.42–0.88)	0.79 (0.50–1.26)
≥ 12	0.09 (0.05–0.18)	0.18 (0.08–0.39)	0.48 (0.27–0.85)	0.55 (0.28–1.10)
Index of goods^b^ (quintiles)	p = 0.001^c^	p = 0.010^c^	p = 0.045^c^	p = 0.819^c^
1st (poorest)	Ref.	Ref.	Ref.	Ref.
2nd	0.63 (0.43–0.93)	0.67 (0.43–1.06)	0.83 (0.57–1.22)	0.90 (0.60–1.35)
3rd	0.79 (0.53–1.17)	0.85 (0.53–1.35)	0.84 (0.56–1.28)	0.94 (0.61–1.44)
4th	0.52 (0.36–0.75)	0.64 (0.41–1.01)	0.73 (0.51–1.06)	0.84 (0.55–1.29)
5th (richest)	0.29 (0.19–0.43)	0.39 (0.24–0.65)	0.60 (0.42–0.85)	0.78 (0.50–1.23)
Number of residents	p = 0.983^c^	p = 0.184^c^	p = 0.239^c^	p = 0.203^c^
1	Ref.	Ref.	Ref.	Ref.
2	0.90 (0.55–1.47)	1.22 (0.76–1.97)	0.92 (0.49–1.72)	1.04 (0.56–1.90)
3	0.93 (0.52–1.67)	1.80 (0.97–3.35)	0.97 (0.52–1.81)	1.23 (0.68–2.23)
4	0.89 (0.52–1.53)	1.86 (1.05–3.30)	0.88 (0.47–1.65)	1.18 (0.67–2.10)
≥ 5	0.93 (0.51–1.71)	1.91 (1.08–3.36)	0.69 (0.35–1.85)	0.94 (0.51–1.74)
Employment	p = 0.007^c^	p = 0.283^c^	p = 0.001^c^	p = 0.023^c^
No	Ref.	Ref.	Ref.	Ref.
Yes	0.71 (0.56–0.90)	0.86 (0.65–1.14)	0.61 (0.47–0.81)	0.69 (0.50–0.94)
Living with a partner	p < 0.001^c^	p = 0.469^c^	p = 0.372^c^	p = 0.949^c^
No	Ref.	Ref.	Ref.	Ref.
Yes	0.99 (0.71 - 1.39)	0.88 (0.62–1.26)	1.10 (0.88–1.39)	1.01 (0.76–1.34)
% of time living in the rural area	p = 0.039^d^	p = 0.055^c^	p = 0.954^c^	p = 0.946^c^
Less than 50%	Ref.	Ref.	Ref.	Ref.
50% to 99%	1.96 (1.34–2.85)	1.48 (1.02–2.14)	1.03 (0.75–1.41)	0.95 (0.67–1.34)
Up to 100%	1.61 (1.16–2.24)	1.35 (1.00–1.82)	0.97 (0.74–1.28)	0.98 (0.75–1.29)
High blood pressure	p < 0.001^c^	p = 0.094^c^	p < 0.001^c^	p = 0.001^c^
No	Ref.	Ref.	Ref.	Ref.
Yes	1.80 (-1.47–2.20)	1.22 (0.96–1.54)	2.36 (1.81–3.09)	1.80 (1.30–2.49)
Diabetes	p < 0.001^c^	p = 0.312^c^	p < 0.001^c^	p = 0.003^c^
No	Ref.	Ref.	Ref.	Ref.
Yes	1.89 (1.39–2.57)	1.18 (0.85–1.66)	2.66 (1.85–3.81)	1.93 (1.28–2.93)
High cholesterol	p < 0.001^c^	p = 0.027^c^	p < 0.001^c^	p = 0.010^c^
No	Ref.	Ref.	Ref.	Ref.
Yes	1.99 (1.55–2.54)	1.39 (1.04–1.85)	2.33 (1.80–3.03)	1.46 (1.10–1.94)
Cardiovascular diseases^e^	p < 0.001^c^	p = 0.172^c^	p < 0.001^c^	p = 0.195^c^
No	Ref.	Ref.	Ref.	Ref.
Yes	2.86 (-2.08– -1.66)	1.51 (0.83–2.75)	3.02 (-2.30–4.46)	1.42 (0.82–2.46)
Respiratory diseases	p = 0.126^c^	p = 0.223^c^	p = 0.002^c^	p = 0.013^c^
No	Ref.	Ref.	Ref.	Ref.
Yes	1.40 (0.90–2.18)	1.33 (0.83–2.13)	1.88 (1.30–2.73)	1.74 (1.13–2.68)
Rheumatic diseases	p = 0.007^c^	p = 0.073^c^	p < 0.001^c^	p < 0.001^c^
No	Ref.	Ref.	Ref.	Ref.
Yes	1.84 (1.20–2.83)	1.48 (0.96–2.29)	3.15 (2.22–4.48)	2.86 (1.93–4.24)

^a^ Ordinal logistic regression adjusted for sex, age, race, education level.

^b^ Index of goods by principal components, employment, number of residents in the household, living with a partner, percentage of time living in the rural area, work, and number of residents.

^c^ P-value for heterogeneity.

^d^ P-value for linear trend.

^e^ Variable with most missing data.

^f^ The odds ratio is constant when moving from one category to another and its interpretation is the risk of moving from the categories of better perception of quality of life and satisfaction with health toward the worst categories of response.

In addition, the chance of presenting lower satisfaction with health was higher among women (p = 0.001) and it also increased linearly as age increased (p = 0.001), being it 126% higher in older adults than in younger individuals. Individuals who had a job were less likely to have a lower satisfaction with their health (95%CI 0.50–0.94). Regarding the presence of diseases, all evaluated diseases were significantly associated with lower satisfaction with health, except for cardiovascular diseases (Table 3).

## DISCUSSION

In this study, we investigated quality of life and its demographic and socioeconomic determinants in the rural population of the city of Pelotas, Brazil, where 23% of the individuals reported having a fair, poor, or very poor overall QOL and 26% reported not being satisfied with their health.

Regarding the determinants of QOL in the rural population of Pelotas, we highlight the negative association between QOL in most domains and females, which has been previously observed in rural and urban areas in Brazil^8^. The greater exposure of women to some factors assessed in the physical and psychological domains, such as drug consumption^14^, depression^15^, and other common psychiatric disorders^16^, has already been observed in the urban area of Pelotas, and it may also be related to QOL in rural areas. Consistently, women in the rural area of Pelotas were more likely to report poorer satisfaction with their health. The presence of women in the rural economy is strongly marked by the sexual division of labor, so that their occupations in rural areas involve not well-paid functions. Activities with little or no remuneration, such as those for family self-consumption, childcare, and care of other family members, as well as gardening and small animal care, can contribute to women having worse QOL in most domains evaluated. The environment domain also presented this same relation for women, possibly because of some aspects related to home environment, recreation or leisure, social and health care, and financial resources^17^.

Rural women have their lives strongly marked by the characteristics of the place where they live, which makes the reflection on their health situation challenging. The health of these women is directly related to living and working conditions, which produce risks such as illness and health problems. These vulnerabilities are due to several factors; among them, we can mention inequality in gender, race, and social class relations related to the social determinants of health conditions, such as diseases related to work, sexual violence, and mental illness^17^. The knowledge on these characteristics implies in the identification of situations that negatively affect the physical, mental, environmental, and health conditions of women, which would allow the development of policies that can contribute to improve the health status and quality of life of women.

The increase in age was also an important factor of vulnerability in the rural area of Pelotas, being related to worse QOL averages for the physical, psychological, and social relation domains and overall perception of QOL. Limitations in work and leisure, aesthetic changes, development of diseases, dependence on other individuals for relationships, and age-related prejudices are factors considered as a consequence of the aging process and could negatively impact QOL^18^ ^,^ ^19^.

Socioeconomic determinants play a relevant role in the context of health behaviors^19^ among persons of lower socioeconomic level and lower education level^19^ ^,^ ^20^. In this sense, we observed a negative association between education level and QOL scores for the physical and psychological domains, as well as perception of overall QOL. The effect of low education level on the acquisition of negative health habits, such as a higher prevalence of sedentary lifestyle, lower consumption of fresh fruits and vegetables, higher consumption of alcoholic beverages, and a higher prevalence of smoking have been described in urban areas^21^. It is possible that the high level of illiteracy in rural areas^22^, also evidenced in this study, may be related to worse health behaviors and, consequently, worse QOL. This highlights the importance of improving the access and quality of education in rural areas, since education level is an important health indicator, which may negatively impact the QOL of this population.

In addition, individuals with lower socioeconomic status had lower QOL scores for all domains and worse perception of overall QOL. Consistently, employed participants had better QOL averages for most domains and a greater chance of having a better satisfaction with their health. Socioeconomic factors reflected in, for example, the number of medical and dental appointments^21^, which can have a direct impact on health. Moreover, employment influences the living standards, behavior, and health of the population, so that higher income, higher education level, and greater health care are observed in employed individuals^23^. Socioeconomic indicators are likely to behave similarly within rural and urban populations so that the results sampled reflect the inequalities related to the demographic, social, cultural, and health characteristics in the general population, which indicates that poorer individuals and those with a lower education level are always among the most vulnerable groups.

Among all the outcomes, the variable of race was only associated with perception of overall QOL. Individuals who self-reported as non-white had a greater chance of having a worse perception of QOL. In Brazil, higher mortality and morbidity rates and lower life expectancy are observed in non-white individuals. Furthermore, this group of individuals also have lower financial conditions and less access to health and knowledge-related resources, in this way they have a disadvantage throughout their lives, both in rural and urban areas, in relation to social and health inequalities in Brazil^6^ ^,^ ^11^.

Living with a partner was associated with better QOL in the psychological domain. The results of the literature do not show a clear direction regarding the mental health of married individuals. Both results of better and worse mental health have already been observed^24^ ^,^ ^25^. However, lower mortality and morbidity rates have been associated with being married in other studies^26^ ^,^ ^27^. In this study, the increase in the number of individuals living in the same household was associated with a decreased average QOL in the domain of social relations. The decrease in the privacy of the residents, both in the aspects related to sexual activity and in the social relations or supports given by the greater number of persons living in the same household, could explain such finding^28^.

In relation to the specific characteristics of rural areas, we highlight the geographical distance from large urban centers and the difficulty in the access to food, employment opportunities, and health services^25–30^. In addition, although there are schools located in the rural area, distance and difficult in movement could be factors that hinder the access of students to the classroom. As a consequence, the association between the greater percentage of time living in the rural area and the lower chance of reporting better QOL may be explained by the scarce infrastructure available in rural areas. Further studies, conducted in other rural areas, are important to confirm these findings, as well as explore possible causes.

In relation to the presence of diseases such as HBP, diabetes, high cholesterol, and cardiovascular, respiratory, and rheumatic diseases, the decrease in the scores of the physical domain was associated with the presence of all diseases, which is in accordance to that observed in rural areas of Turkey^30^ and may reflect disabilities and limitations in individuals for work and leisure activities^6^.

This study presents a limitation, which is intrinsic to its sampling process; that is, individuals living far from the area of selected households may present health profiles and lifestyles different from those evaluated, which could modify the observed results. Furthermore, the period of study included months of greater harvest of some crops, and we could not interview the individuals involved in exhaustive harvest work, who could report a worse QOL. On the other hand, we highlight the large sample size and the low percentage of losses and refusals. Regarding QOL, we highlighted the use of an instrument developed by the World Health Organization (WHO) and validated in Brazil. In addition, this study is distinguished by the fact that it is population-based, with a sample of adults living in rural areas. Most studies evaluating QOL are specific to the urban area, with older adults, and performed with persons with morbidities.

Finally, this study presents alarming data regarding an important health marker, poorly explored in rural populations. Approximately a quarter of the population reported a fair, poor, or very poor QOL and little satisfaction with health. In addition to the fact that individuals living in rural areas throughout their life have a poorer QOL, the findings show the importance of implementing health programs that seek to improve the QOL of this population. Furthermore, this work highlights the vulnerability of three important groups: women, poorer individuals with a lower education level, and older adults. The QOL as a health indicator generates information that can be used to screen and identify the health needs of a population. The information presented here can be used in specific public health programs for inhabitants of rural areas of Brazil, considering the peculiarities of the place regarding the infrastructure and the health needs of this population.
